# A review of menopause nomenclature

**DOI:** 10.1186/s12978-022-01336-7

**Published:** 2022-01-31

**Authors:** Ananthan Ambikairajah, Erin Walsh, Nicolas Cherbuin

**Affiliations:** 1 Centre for Research on Ageing, Health and Wellbeing, Australian National University, Canberra, ACT 2601 Australia; 2 Discipline of Psychology, Faculty of Health, University of Canberra, Building 12, 11 Kirinari Street, Canberra, ACT 2617 Australia

**Keywords:** Menopause, Nomenclature, STRAW, WHO

## Abstract

Menopause nomenclature varies in the scholarly literature making synthesis and interpretation of research findings difficult. Therefore, the present study aimed to review and discuss critical developments in menopause nomenclature; determine the level of heterogeneity amongst menopause definitions and compare them with the Stages of Reproductive Aging Workshop criteria. Definitions/criteria used to characterise premenopausal and postmenopausal status were extracted from 210 studies and 128 of these studies were included in the final analyses. The main findings were that 39.84% of included studies were consistent with STRAW classification of *premenopause*, whereas 70.31% were consistent with STRAW classification of *postmenopause*. Surprisingly, major inconsistencies relating to premenopause definition were due to a total lack of reporting of any definitions/criteria for premenopause (39.84% of studies). In contrast, only 20.31% did not report definitions/criteria for postmenopause. The present findings indicate that there is a significant amount of heterogeneity associated with the definition of *premenopause*, compared with *postmenopause*. We propose three key suggestions/recommendations, which can be distilled from these findings. Firstly, premenopause should be transparently operationalised and reported. Secondly, as a minimum requirement, regular menstruation should be defined as the number of menstrual cycles in a period of at least 3 months. Finally, the utility of introducing normative age-ranges as supplementary criterion for defining stages of reproductive ageing should be considered. The use of consistent terminology in research will enhance our capacity to compare results from different studies and more effectively investigate issues related to women’s health and ageing.

**Supplementary Information:**

The online version contains supplementary material available at 10.1186/s12978-022-01336-7.

## Plain Language Summary

The meaning of *menopause* is widely understood, but often imprecisely defined in research. The present findings revealed that there is a significant amount of heterogeneity associated with the definition of *premenopause*, compared with *postmenopause*. Three key suggestions/recommendations can be distilled from these findings. Firstly, premenopause should be transparently operationalised and reported. Secondly, as a minimum requirement, regular menstruation should be defined as the number of menstrual cycles in a period of at least 3 months. Finally, the utility of introducing normative age-ranges as supplementary criterion for defining stages of reproductive ageing should be considered. The use of consistent terminology in research will enhance our capacity to compare results from different studies and more effectively investigate issues related to women’s health and ageing.

**Supplementary Information:**

The online version contains supplementary material available at 10.1186/s12978-022-01336-7.

## Introduction

Menopause is a critical stage of female reproductive ageing and health, with important implications relating to fat mass and its distribution [1], dyslipidemia [2] and neurodegeneration [3, 4]. In this context, it is likely that some of the biological changes co-occurring with menopause, contribute to the well-documented higher risk of dementia in women [5], as well as the observed increase in cardiovascular disease whose pattern becomes more similar to that of men at older ages despite its lower prevalence at younger ages [6, 7]. However, the contributions of menopause to health have been historically understudied in the context of ageing [8]. For example, over a period of 23 years (1995–2017), peer-reviewed neuroimaging articles which focused on menopause only accounted for approximately 2% of the ageing literature [8]. There are many possible explanations (including sex biases in research), however, a critical challenge for menopause research has been the operationalisation of menopause nomenclature.

The meaning of *menopause* is widely understood, but often imprecisely defined in research. The standards for defining menopause nomenclature, such as *premenopause* and *postmenopause* vary substantially across publications. Although, the precise extent of this heterogeneity remains to be established—perhaps because the extant literature on this topic may be too large to systematically review—it is clear that such variability across studies makes the synthesis and comparison of findings difficult. In recognition of this issue, there have been a number of attempts by international experts to collaboratively develop a comprehensive standardised set of criteria to describe terminology associated with menopause [9–14]. Whilst promising developments have been made in recent decades, a follow-up investigation regarding the frequency and consistency of uptake and use of the proposed criteria have not been adequately investigated. Therefore, the degree to which standardised criteria have been successfully implemented in publications relating to menopause research remains unknown.

To address this gap we have leveraged on our recent systematic review with meta-analysis focused on fat mass differences between premenopausal and postmenopausal women, which included 210 studies consisting of 1,052,391 women, by extracting definitions used to characterise premenopausal and postmenopausal status in a broad cross-section of peer-reviewed literature [1]. The present study aims to first review and discuss critical developments in menopause nomenclature, with a particular emphasis placed on the implications that current criteria have for menopause research. Then, to assess the level of heterogeneity in menopause nomenclature identified through our previous systematic review [7]. Finally, to contrast the extracted definitions against the Stages of Reproductive Aging Workshop (STRAW) criteria [11, 13, 14]^.^

### WHO (1981–1999)

**Fig. 1:**
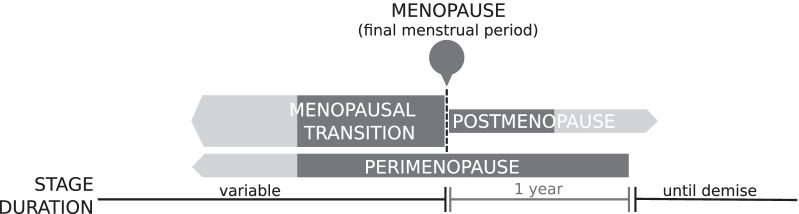
Visual representation of the relationship between different time periods surrounding menopause as established by a World Health Organization Scientific Group on Research in the Menopause. Figure is a modification of work found in World Health Organization [9]

According to the more recently established guidelines by a World Health Organization (WHO) “Scientific Group on Research in the Menopause”, natural menopause is defined as the permanent cessation of menstruation resulting from the loss of ovarian follicular activity [9, 10]. Furthermore, natural menopause is deemed to have occurred after 12 consecutive months of amenorrhea, for which no other obvious pathological or physiological causes could be determined. As seen in Fig. 1, menopause occurs at the final menstrual period (FMP), which can only be known with certainty retrospectively, a year or more after the event. Induced menopause, however, is defined as the cessation of menstruation following either surgical removal of both ovaries (i.e. oophorectomy), or iatrogenic ablation of ovarian function (i.e. chemotherapy or irradiation).

**Fig. 2:**
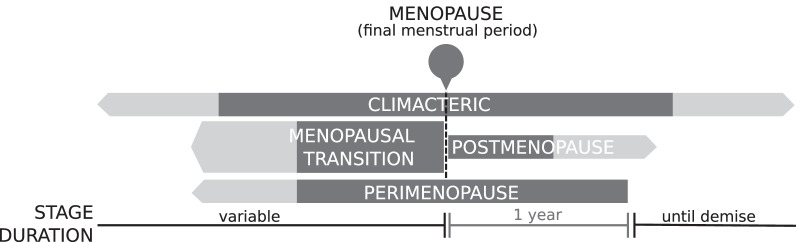
Updated visual representation of the relationship between different time periods surrounding menopause, which includes the term *Climacteric* as defined by The Council of Affiliated Menopause Societies. Figure is a modification of work found in Utian [13]

The WHO (1996) highlighted that *premenopause* was often used ambiguously by researchers, either to refer to the 1 or 2 years immediately before menopause or alternatively, to encompass the entire reproductive period up to the FMP, which was the recommended use of the term. Other critical stages defined by the WHO included *postmenopause* (i.e. the period following the FMP regardless of whether menopause was induced or spontaneous); perimenopause (i.e. the period immediately prior to the FMP when endocrinological, biological and clinical features of approaching menopause commence, as well as the first year after menopause); and the menopausal transition (i.e. the period of time before FMP, when variability in the menstrual cycle is usually increased). Finally, it was strongly recommended that the term *climacteric*, which was previously used interchangeably with *perimenopause*, should be abandoned to avoid confusion. However, due to widespread popularity and the prevailing use of the word, *climacteric* was reinstated by The Council of Affiliated Menopause Societies (CAMS) in 1999 and was defined as a phase which incorporates perimenopause, but extends for a longer variable period before and after perimenopause and marks the transition from the reproductive to non-reproductive states (Fig. 2) [13].

### STRAW (2001)

**Fig. 3:**
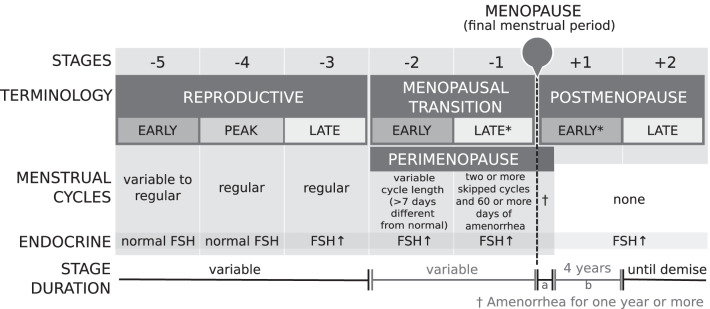
STRAW staging system. *Stages most likely to be characterised by vasomotor symptoms; FSH, follicle stimulating hormone; ↑, elevated. Figure is a modification of work found in Soules et al. [14]

The nomenclature established thus far facilitated a scientific consensus for describing female reproductive ageing, however, there were still limitations that needed to be addressed. For example, the WHO and CAMS definitions had vague starting points and used terms such as premenopause, perimenopause, menopausal transition and climacteric which, to some extent, had overlapping time periods. This lack of clear, objective criteria to describe the stages of female reproductive ageing led to the Stages of Reproductive Ageing Workshop (STRAW) in 2001. The ensuing STRAW criteria separated the stages of female reproductive ageing into seven distinct segments (Fig. 3), with a particular focus on healthy women undergoing natural menopause. Furthermore, menstrual cycles, endocrine/biochemical factors, signs/symptoms in other organ systems, and uterine/ovarian anatomy were used to define the stages of female reproductive ageing.

Within the STRAW criteria, menopause is central to the staging system and was labelled as point zero (0). There are five stages preceding the FMP (− 5 to − 1) and two following it (+ 1 to + 2). Stages − 5 to − 3 encompassed the Reproductive Interval; − 2 to − 1 reflected the Menopausal Transition; and + 1 to + 2 defined Postmenopause [14]. The menopausal transition (− 2 to − 1) began with a variation in menstrual cycle length and rise in follicle stimulating hormone (FSH) and ended with the FMP. Early postmenopause (+ 1) was defined as within 5 years since the FMP and was further subdivided into segments ‘a’; the first 12 months after the FMP and ‘b’; the following 4 years. Whereas late postmenopause (+ 2) was defined as having a variable duration since it ended with a woman’s death. Finally, the STRAW criteria defined perimenopause (− 2 to + 1a) as ending 12 months after the FMP. Furthermore, it was suggested that the terms perimenopause and climacteric should be synonymous in meaning and used with patients or the public, but not in scientific papers, in accordance with the WHO recommendations.

Importantly, the validity and reliability of the STRAW recommendations has been evaluated and was broadly supported by the ReSTAGE Collaboration, which conducted empirical analyses on four cohort studies including the TREMIN study, the Seattle Midlife Women’s Health Study, the Study of Women’s Health Across the Nation (SWAN) and the Melbourne Women’s Midlife Health Project [12, 15, 16]. However, particular limitations have also been noted and modifications to the STRAW criteria were suggested by the ReSTAGE collaboration. In particular, when the STRAW criteria were first established, there was a lack of multiethnic cohort studies available, which limited the generalisability of the staging system to diverse populations [11]. Furthermore, the initial STRAW criteria only considered FSH as a biomarker, with relatively little clarification about the precise timing of change in FSH levels or quantitative criteria for FSH, due to insufficient data [11]. As a result, the initial STRAW criteria focused primarily on menstrual bleeding patterns and qualitative FSH levels. Other important limitations of the original STRAW criteria included their exclusive applicability to healthy women, with explicit recommendations against applying the criteria to women who either (i) smoked, (ii) had a BMI greater than 30 $\mathrm{kg}/{\mathrm{m}}^{2}$ or less than 18 $\mathrm{kg}/{\mathrm{m}}^{2}$, (iii) engaged in heavy exercise (greater than 10 h per week of aerobic exercise), (iv) had chronic menstrual cycle irregularity, (v) had a prior hysterectomy, (vi) had abnormal uterine anatomy (e.g. fibroids) or (vii) had abnormal ovarian anatomy (e.g. endometrioma).

### STRAW + 10 (2011)

In 2011, the STRAW + 10 criteria [11] were established to reflect significant advances in the field of female reproductive ageing and to provide updated recommendations that addressed certain limitations present in the initial staging criteria.

**Fig. 4:**
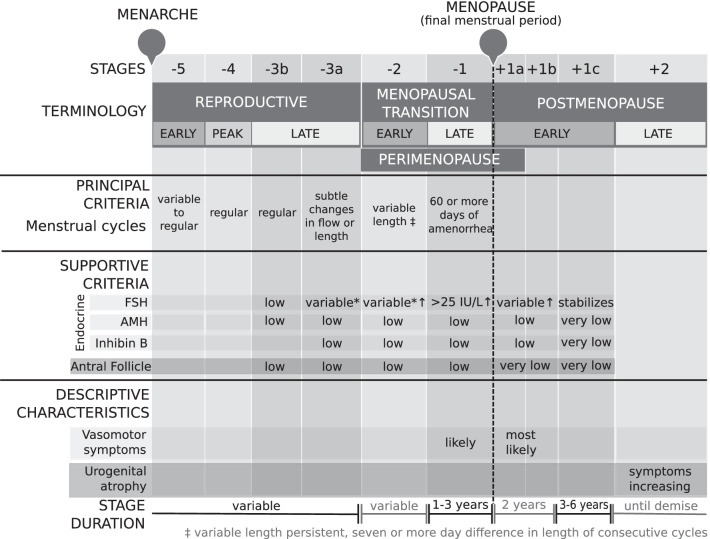
STRAW + 10 staging system. *, blood drawn on cycle days 2–5; FSH, follicle stimulating hormone; AMH, anti-mullerian hormone; ↑, elevated. Figure is a modification of work found in Harlow et al. [11]

The STRAW + 10 staging system suggested that the late reproductive stage (− 3) should be subdivided into two stages (− 3b and − 3a) based on menstrual cycle characteristics and FSH levels (Fig. 4). This was done to recognise subtle changes in menstrual cycle flow and also shorter cycle lengths in stage − 3a, in addition to an increased variability in FSH levels [11]. Secondly, the new recommendations incorporated the suggestions provided by the ReSTAGE Collaboration, which proposed that more precise menstrual cycle criteria should be used to describe the early (− 2) and late (− 1) menopausal transition, in addition to the quantification of FSH levels in late menopausal transition [4]. Specifically, the early menopausal transition (− 2) was discernible from the late reproductive stage (− 3a) due to an increased variability in menstrual cycle length (defined as a difference of 7 days or more in length of a menstrual cycle that is persistent i.e. reoccurs within 10 cycles of the first variable length cycle). Furthermore, late menopausal transition (− 1) was marked by an interval of amenorrhea greater or equal to 60 days, in addition to an increased FSH level greater than 25 IU/L [11, 12]. Finally, early postmenopause (+ 1) was further subdivided into three stages (+ 1a, + 1b, + 1c) to account for the continual increase in FSH and decrease in estradiol for 2 years after FMP, whereby + 1a corresponded with 12 months after FMP i.e. end of perimenopause and + 1b referred to the year prior to the stabilisation of high FSH and low estradiol levels (+ 1c).

The STRAW + 10 staging system has been found to be applicable to most women regardless of age, demographic, body mass index (BMI) or lifestyle characteristics [11]. However there are still significant areas of scientific research that need to be prioritised to strengthen future criteria including (i) the use of standardised assays for key biomarkers (e.g. Anti-Mullerian hormone), (ii) further empirical analysis across multiple cohorts to specify menstrual cycle criteria for the late reproductive stage, and (iii) further research aimed at better understanding reproductive ageing in women who have had either the removal of a single ovary and/or a hysterectomy, chronic illness such as HIV infection, cancer treatment, polycystic ovary syndrome or premature ovarian failure [11]. Another critical limitation of the STRAW + 10 criteria is that they do not apply to women who are using exogenous hormones, such as hormone replacement therapy (HRT). Likely because HRT use may confound the accurate classification of women into distinct reproductive stages. This is a key consideration that needs to be appropriately accounted for in studies that are interested in investigating varying outcomes in women at different stages of reproductive ageing.

Despite these limitations, the STRAW criteria has significantly advanced our understanding of women’s health and is widely considered the current gold standard for defining terms related to female reproductive ageing. However, the uptake and use of the STRAW criteria in publications relating to menopause research remains unknown and is addressed next.

## Methods

The definitions of premenopausal and postmenopausal women were extracted from the 210 studies (Additional file 1: Table 1, Additional file 2: Table 2) [17–134, 134–168, 168–225, 236] that were eligible for inclusion in the meta-analysis from a previous systematic review, which aimed to identify all peer-reviewed articles reporting on changes in fat mass around menopause [1]. Given that the focus of the present study is the relationship between definitions used in the current literature and the STRAW criteria, only studies published 4 years after the establishment of the STRAW criteria in 2001 (i.e. 2005 onwards) have been included in the analysis. The 4-year lag time was implemented to conservatively account for the ‘study inception to publication’ timeframe, which may have limited the ability for certain studies published between 2001 and 2005 to effectively implement the STRAW criteria. Similarly, longitudinal studies, which had baseline assessments prior to 2005, were excluded. Therefore, 128 studies were included in the final analyses.

### Protocol and registration

The methodology of the initial meta-analyses is reported elsewhere in detail [1] and was pre-registered in the PROSPERO database (CRD42018100643), which can be accessed online (http://www.crd.york.ac.uk/PROSPERO/display_record.php?ID=CRD42018100643).

### Search string

The PubMed database was used to conduct a systematic search and retrieve all studies that reported fat mass differences in quantity or distribution between premenopausal and postmenopausal women. The following search string was used: (“adipose tissue” OR “adiposity” OR “subcutaneous fat” OR “obesity” OR “overweight” OR “body weight” OR “body fat distribution” OR “body mass index” OR “BMI” OR “DEXA” OR “DXA” OR “dual energy x-ray absorptiometry” OR “waist to hip ratio” OR “waist-hip ratio” OR “waist circumference” OR “x-ray computed tomography” OR “computed tomography” OR “CT scan” OR “caliper” OR “skinfold” OR “skin fold” OR “abdominal MRI” OR “abdominal magnetic resonance imaging” OR “intra-abdominal fat”) AND (“menarche” OR “pre-menopause” OR “premenopause” OR “pre-menopausal” OR “premenopausal” OR “reproductive” OR “menopausal transition”) AND (“post-menopause” OR “postmenopause” OR “post-menopausal” OR “postmenopausal” OR “non-reproductive”). PubMed filters were used to exclude non-human and non-English studies. No time restrictions were applied to the literature search, which was conducted in May 2018.

### Inclusion and exclusion criteria

Studies that investigated both healthy premenopausal and healthy postmenopausal women were included, whereas studies that (i) exclusively investigated clinical/pathophysiological populations or (ii) had fewer than 40 participants were excluded.

### Data extraction

Available definitions/criteria used to describe premenopausal and postmenopausal women were extracted from each study. Where data was missing or unclear, authors were contacted via email to obtain relevant information. All data from included articles was double extracted by two authors (AA and EW) to avoid transcription errors with any disagreement resolved by consensus.

### Quality assessment

The quality of included studies was independently assessed by two authors (AA and EW), using an adapted version of the Newcastle–Ottawa Scale (NOS) [226]. More information on the quality of included studies can be found in our recent systematic review with meta-analysis [1]. In short, the NOS for cohort studies utilised three categories to evaluate individual study quality including (1) the selection of participants, (2) the comparability of groups and (3) the assessment/ascertainment of the outcome of interest. Notably, a clear definition of premenopausal and postmenopausal women was included as a criterion when assessing study quality, specifically for the comparability of groups. Any discrepancy in quality assessment was resolved by consensus. If consensus decisions were not possible a third rater was used.

## Results

**Fig. 5:**
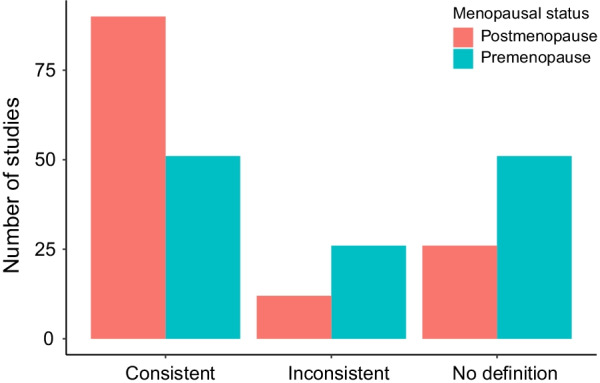
Consistency of definitions with STRAW criteria

The raw extracted definitions for studies are presented in Additional file 1: Table 1 and Additional file 2: Table 2. The consistency of definitions with STRAW criteria for included studies is presented in Fig. 5.

### Premenopausal women

#### Cycle regularity

A total of 41 studies included the criterion *regular menstruation*, three included *regular menstruation in the last 5 years*, 1 included *regular menstruation in the past 2 years* and 1 included *regular menstruation in the past year*. Therefore, 46 studies (35.94%) were consistent with STRAW classification of premenopause, based on menstrual cycles.

Two studies used *still cycling*, 2 used *no increase in cycle irregularity* and 2 used *no change in flow* when characterising premenopausal women. Cycle regularity was further quantified by the use of cycles per month(s) or cycles per year(s). Three studies included the criteria *one menstruation in the past 33 days*, 2 included *two menstruations in the last 3 months*, 1 included *at least one menstruation in the last 3 months*, 1 included *11–13 cycles per year*, 1 included *8 menses in the last year*, 2 included *one menstrual cycle in the last 12 months* and 1 included *one menstrual cycle in the last 2 years*. One study identified premenopause as *the whole reproductive period up until menopause*.

#### Hormone levels

Six studies 4.69% used FSH levels as one of the criteria, consistent with STRAW classification of premenopause, based on hormone levels. Of these 6 studies, 1 used *regular menstruation* as an additional criterion, whereas the other 5 attempted to quantify cycle regularity. The threshold for FSH levels ranged from less than 20 IU/L to less than 40 IU/L.

#### Age

Four studies included women over a specific age ranging from 40 to 44. However all 4 studies also included other subcategories such as *regular menstruation*. Two studies used age brackets that included *25–45*, and *45–55*. Ten studies included women who were less than a specific age, which ranged from 35 to 55 years. Of these 3 studies used *age* as the only criterion to define premenopause. One study included *age* as a subcategory of their definition, however, did not define it precisely.

#### Not postmenopausal or pregnant

Five studies included *no criteria for postmenopause*, 4 included *no symptoms of menopause*, 4 included *no climacteric complaints*, 3 included *no HRT use* and 3 included *no hysterectomy or ovaries removed* as criteria for categorising premenopause. One study used pregnancy as a criterion for defining premenopause.

#### No definition

Of the 128 studies included, 51 (39.84%) did not report definitions/criteria for premenopause.

### Postmenopausal women

#### Amenorrhea or the final menstrual period (FMP)

Eighty studies included the criterion *at least 12 months of amenorrhea*, 1 included *less than 2 years from the FMP*, 1 included *1–5 years since the FMP*, 1 included *0–6 years after the FMP*, 1 included *greater than 1 but less than 7 years of amenorrhea*, 1 included *greater than 2 but less than 7 years amenorrhea* and 2 included *2 years after the FMP*. Therefore, 87 studies (67.97%) were consistent with STRAW classification of postmenopause, based on menstrual cycles.

Two studies included *at least 6 months of amenorrhea* and 1 included *at least 11 months of amenorrhea*. Three studies included the term *no menstrual cycles or periods or no menstrual bleeding* however, further detail regarding the duration of amenorrhea was not provided.

#### Hormone levels

Fourteen studies (10.94%) used FSH levels as a criterion, consistent with STRAW classification of postmenopause, based on hormone levels. Of these 11 studies used menstrual criteria consistent with STRAW, 2 used hormonal criterion alone and 1 included *no menstrual bleeding*. For hormone thresholds, of the 14 studies, 8 used the threshold for FSH levels *as greater than 30 IU/L* and 2 used *greater than 40 IU/L*. One study did not report FSH thresholds, whereas the remaining 3 studies had FSH levels that included *greater than 20 IU/L, greater than 55 IU/L* and *between 22 to 138 IU/L*. Two studies used estradiol levels with thresholds ranging from *less than 20 pg/mL* to *less than 50 pg/mL*. One study also used Luteinizing Hormone (LH) levels *greater than 30 IU/L*.

#### Natural or surgical menopause

Twelve studies specifically stated *natural menopause*, 3 stated *no surgical removal of ovaries and/or uterus* and 2 stated *not due to surgery or any other biological or physiological causes*. Twelve studies included the criteria *bilateral oophorectomy*, 2 included *hysterectomy* and 1 included *cessation of menses induced by surgery*.

#### Age

Twelve studies included women over a specific age, ranging from *40 to 55*. Of these 2 studies used age as the only criterion to define postmenopausal women.

#### Hormone replacement therapy (HRT)

Five studies included *women not taking HRT*, whereas 4 studies included *women taking HRT*, and 1 study included *women taking ovarian suppressing drugs or contraception eliminating menstruation*.

#### No definition

Of the 128 studies included, 26 (20.31%) did not report any definitions/criteria for postmenopause.

## Discussion

To our knowledge, this review is the first to assess the uptake and use of the STRAW criteria by extracting definitions used to characterise premenopausal and postmenopausal status in a broad cross-section of peer-reviewed literature from our recent systematic review with meta-analysis [1]. The main findings were that 39.84% of included studies were consistent with STRAW classification of *premenopause*, whereas 70.31% were consistent with STRAW classification of *postmenopause* (Fig. 5). Furthermore, 39.84% did not report definitions/criteria for premenopausal women, whereas, 20.31% did not report definitions/criteria for postmenopausal women.

**Fig. 6:**
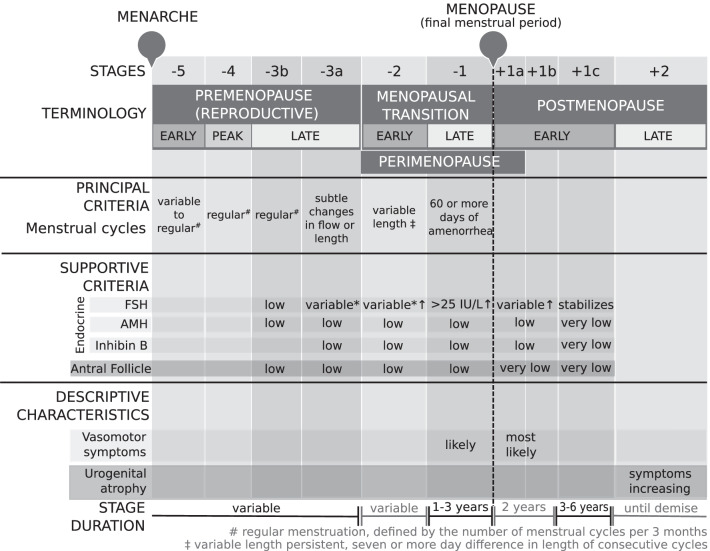
Recommended revision to the STRAW + 10 staging system to include the transparent operationalisation of *premenopause* and define *regular menstruation* as the number of menstrual cycles per 3 months, as a minimum requirement, which would be a practical reporting timeframe both clinically and for women to recall accurately. *, blood drawn on cycle days 2–5; FSH, follicle stimulating hormone; AMH, anti-mullerian hormone; ↑, elevated. Figure is a modification of work found in Harlow et al. [11]

For menstrual cycle variability, 35.94% of studies were consistent with STRAW classification of *premenopause* and 67.97% for *postmenopause*. Notably, STRAW + 10 later distinguished menstrual cycle variability as the most important criteria for the reproductive staging system [11], which is reflective of its use in the literature. For *postmenopause*, the current results reflect a conceptualisation consistent with the STRAW criteria, which require the relationship between the FMP and start of *postmenopause* to be explicitly defined. However, this same level of consistency was not observed for *premenopause*. One possible explanation relates to the term *premenopause* not having been explicitly used in the STRAW criteria [11, 14]. Instead, it is inferred to be synonymous with *reproductive stage*. Given its wide clinical and scientific use, our recommendation is that the transparent operationalisation of *premenopause* may improve the consistency and application of the STRAW criteria (Fig. 6). Another possibility is the degree of uncertainty regarding the precise meaning of *regular menstruation*. Specifically, 14.29% of studies that defined *premenopause* attempted to quantify regular menstruation as the number of menstrual cycles per days, month(s) or year(s). This uncertainty may reflect a key limitation of the STRAW [14] and more recent STRAW + 10 [11] criteria, which principally describe the reproductive period as having *regular menstrual cycles*, with no guidelines provided regarding the interpretation of *regular*. Moreover, previous research has demonstrated the lack of clear clinical definitions for reproductive stages can significantly decrease the accuracy of participant’s self-report [227]. Since menstrual cycles can be skipped due to reasons unrelated to menopause including extreme exercise, pregnancy, weight fluctuations or illness it would be highly preferable if *regular menstruation* was specifically and consistently defined for a defined period. We recommend that defining regular menstruation as the number of menstrual cycles per 3 months, as a minimum requirement, would be a practical reporting timeframe both clinically and for women to recall accurately (Fig. 6).

For hormone levels, 4.69% of studies were consistent with STRAW classification of premenopause and 10.94% for postmenopause. STRAW + 10 later distinguished hormone levels as a supportive criterion for the reproductive staging system given the lack of international standardisation of biomarker assays as well as their cost and/or invasiveness and inequity across low-socioeconomic countries [11]. Notably, Anti-Mullerian hormone (AMH) has emerged as a primary candidate for developing an international standard biomarker since it is detectable in peripheral circulation [228] and does not change in response to an acute endogenous rise in hormones such as FSH and estrogen [229–231]. Whilst promising, insights about staging reproductive ageing can also be drawn from research that aims to predict age of menopause. Unsurprisingly, age is a useful predictor of menopausal status [232], given ageing and menopause co-occur [233]. However, evidence suggests that the combination of hormones, such as AMH and age does not provide a statistically significant improvement to predictions of time to menopause than age alone (Age C-statistic = 84%, 95% CI 83–86%; Age + AMH C-statistic = 86%, 95% CI 85–87%) [232]. These findings indicate that there is utility in introducing normative age-ranges as a supplementary criterion for defining stages of reproductive ageing. Compared with the establishment of standardised biomarker assays, the use of normative age-ranges can be done relatively quickly and reliably, using available evidence from multiple large population studies, such as the UK Biobank study [234]. This need is recognised by the number of studies in this review with a definition that has attempted to use age to further clarify menopausal status (Premenopause: 19.48%; Postmenopause: 11.76%). Moreover, the use of age as an additional component of the supportive criteria for determining reproductive stage becomes further evident when women who use HRT or suffer from chronic illness are considered. For example, a systematic review with meta-analysis of randomised controlled trials showed that the incidence of chemotherapy induced amenorrhea is 61% (95% CI 51–68%) for women with breast cancer [235]. For these women, the current use of principal criteria, which relies solely on menstrual cycles, is inadequate. This emphasises the urgent need to expand the supportive criteria to ensure STRAW + 10 can be utilised by women using HRT or suffering from chronic illness that impacts menstrual cycles.

Altogether, 33.77% of studies that defined premenopause and 11.76% of studies that defined postmenopause used criteria inconsistent with STRAW criteria. The disproportionate use of additional criteria for defining *premenopause* compared with *postmenopause* is further indication that the term *premenopause* is not precisely and systematically defined by the STRAW criteria. This has prompted researchers to use additional/alternative criteria to achieve clarity. Unfortunately, the consequence of non-standardised criteria is increased heterogeneity, which can lead to the synthesis of imprecise estimates. Moreover, of the 128 included studies, 39.84% did not report definitions/criteria for premenopausal women, whereas, only 20.31% did not report definitions/criteria for postmenopausal women. This difference may reflect a belief that the definition/criteria for premenopausal women is widely understood, with no need for further clarification by authors. However, in the context of the findings presented in this review, it is more likely these trends reflect a poor understanding of the term *premenopause* compared with *postmenopause*.

## Conclusion

There is a significant amount of heterogeneity associated with the definition of *premenopause*, compared with *postmenopause*. We propose three key suggestions/recommendations, which can be distilled from these findings. Firstly, premenopause, which is not currently explicitly stated in STRAW or STRAW + 10, should be transparently operationalised and reported. Secondly, as a minimum requirement, regular menstruation should be defined as the number of menstrual cycles in a period of at least 3 months. Finally, the utility of introducing normative age-ranges as supplementary criterion for defining stages of reproductive ageing should be considered. The use of consistent terminology in research will enhance our capacity to compare results from different studies and more effectively investigate issues related to women’s health and ageing.

## Supplementary Information

**Additional file 1: Table 1.** Premenopause definition.

**Additional file 2: Table 2.** Postmenopause definition.

## Data Availability

The data generated or analysed during this study are included in this published article [and its Additional files 1 and 2].
