# Supplementary material for: A review of menopause nomenclature
Source: Reprod Health. 2022 Jan 31;19:29. doi: 10.1186/s12978-022-01336-7 (PMC8805414; doi:10.1186/s12978-022-01336-7)
Supplement: Supplementary file 1 — Additional file 1: Table 1. Premenopause definition. [file 12978_2022_1336_MOESM1_ESM.pdf]

Additional Table 1: Premenopause definition.

Study	Year	Premenopause definition
Abate et al.	2014	Women older than 44 who had regular menstrual cycles
Abdulnour et al.	2012	Two menstruations in the last 3 months, no increase in cycle irregularity in the 12 months before testing and FSH <30IU/L.
Abildgaard et al.	2013	Menstrual bleeding within the last 12 months, and FSH <20IU/L
Adams-Campbell et al.	1996	Unclear
Agrinier et al.	2010	Pregnant, or regular menstruation under 40 years, or irregular menstruation under 40 years, regular menstruation over 40 with no progestin use.
Aguado et al.	1996	Unclear
Akahoshi et al.	2001	Four years before the final menstrual period (FMP), where the FMP is amenorrhea for more than 12 months, except for pregnancy
Albanese et al.	2009	Menstrual histories indicating current and prior menstrual regularity 11 to 13 cycles per year
Allali et al.	2009	Unclear
Aloia et al.	1995	Unclear
Amankwah et al.	2013	Based on age, no hysterectomy, no menopause, no ovaries removed and no symptoms of menopause
Amarante et al.	2011	Regular menstrual cycles and had spontaneous menstrual cycle in the last month
Amiri et al.	2014	Unclear
Angsuwanthana et al.	2007	Women who were older than 40 years and did not have criteria for postmenopausal women
Armellini et al.	1996	Unclear
Arthur et al.	2013	Still menstruating irrespective of the regularities of their menses
Aydin et al.	2010	Women who were not postmenopausal (12 months past final menses), were considered premenopausal
Ayub et al.	2006	Unclear
Bancroft et al.	1996	Regular cycles and there was evidence of ovulation during the month of sampling (plasma progesterone >=10nmol/l) or irregular cycles but had ovulated during the month
Bednarek-Tupikowska et al.	2006	Unclear
Bell et al.	2007	A decision tree including - younger than 55, no stated age at menopause, regulary menstrual bleeding, no use of hormone contraception and FSH levels
Ben-Ali et al.	2016	Unclear
Ben-Ali et al.	2014	Unclear
Ben-Ali et al.	2011	Regular periods in the years preceding their examination
Berg et al.	2004	Regular cycles
Berge et al.	1994	Cycles were regular
Berger et al.	1995	Regular menstruation, lack of menopausal symptoms and normal gonadotropin levels
Berstad et al.	2010	Women who wee still menstruatingand had not taken any hormone therapy before the reference date
Bhagat et al.	2010	Unchanged and regular menstrual pattern during the last 5 years without typical climacteric complaints
Bhurosy et al.	2013	Having regular menstrual bleeding
Blumenthal et al.	1991	Regular menstrual cycle lengths and had not taken hormones orally in the past year and FSH less than 40
Bonithon-Kopp et al.	1990	Not experienced the menopause and that their last menstrual period had occurred in the last three months
Caire-Juvera et al.	2008	Experiencing a mestrual cycle within the past 12 calandar months or having a FSH level < 22 mIU/ml

(continued)

Study	Year	Premenopause definition
Campesi et al.	2016	With regular menstrual cycles (27-29 days)
Carr et al.	2000	No change in the past year in menstrual flow amount, duration or cycle length, as well as no change in regularity since ages 20-35 without hormone use
Castracane et al.	1998	Normal cycling female subjects, with cycle length between 25 and 35 days
Catsburg et al.	2014	Unclear
Cecchini et al.	2012	Women younger than 50
Cervellati et al.	2009	Regular menstrual cycle
Chain et al.	2017	Unclear
Chang et al.	2000	Age less than 48 with regular menstruation
Cho et al.	2008	Unclear
Cifkova et al.	2008	FMP occurred less than 60 days before the interview and FSH $<$ 40IU/L
Copeland et al.	2006	Cycling regularly
Cremonini et al.	2013	Regular menstrual cycle
Cui et al.	2007	Regular menstrual cycle
D'haeseleer et al.	2011	Regular menstrual cycle
Da Camara et al.	2015	Regular menstruation
Dallongeville et al.	1995	Unclear
Dancey et al.	2001	Less than 45 years of age
Davis et al.	1994	Currently have menstrual cycles/periods
De Kat et al.	2017	Women with a currently regular menstrual cycle
Den Tonkelaar et al.	1990	Unclear
Dmitruk et al.	2018	Regularly menstruating women
Donato et al.	2006	Women with no change in menstrual flow or frequency
Douchi et al.	1997	Regular menstruation
Douchi et al.	2002	Regular menstruation
Douchi et al.	2007	Unclear
Dubois et al.	2001	Women who were not amenorrhoeic for at least 12 months
Engmann et al.	2017	Self-reported as pre or peri-menopausal or age $<=$ 55
Ertungealp et al.	1999	Unclear
Feng et al.	2008	Regular menstrual period every 21-40 days without significant changes in the past year
Ford et al.	2005	Menstrual cycle in the last 12 months and not using oral contraceptive pill or other hormone products and not pregnant or lactating
Formica et al.	1995	Unclear
Franklin et al.	2009	Unclear
Friedenreich et al.	2007	Regular menses over the past 12 months pr reported using HRT and we under the age of 46
Friedenreich et al.	2002	Based on age, no hysterectomy, no menopause, no ovaries removed and no symptoms of menopause
Fu et al.	2011	Regular menstruation defines as the 25-35 day interval between menstrual on-set

(continued)

Study	Year	Premenopause definition
Fuh et al.	2003	Regular menstruation
Gambacciani et al.	1999	Regular menstrual cycle
Genazzani et al.	2006	Regular menstrual cycle
Ghosh et al.	2008	Unchanged and regular menstrual pattern during the last 5 years without typical climacteric complaints
Ghosh et al.	2010	Unchanged and regular menstrual pattern during the last 5 years without typical climacteric complaints
Gram et al.	1997	Unclear
Guo et al.	2015	Women younger than 45 years who had not undergone a bilateral oophorectomy
Gurka et al.	2016	Women who had a menstrual period in the past 2 years but denied current menopause
Hadji et al.	2000	Women with regular periods in the year preceding their examination and/or had a serum FSH of <30IU/L and a serum estradiol of >10pg/ml
Hagner et al.	2009	FMP less than 60 days
Han et al.	2006	Unclear
Harting et al.	1984	Not having menses due to hysterectomy without bilateral oophorectomy younger than age 40 or due to lactation
He et al.	2012	Having regular menstrual cycles during recent one year and time since their last menstruation was less than 33 days
Hirose et al.	2003	Unclear
Hjartaker et al.	2005	All women who did not report natural menopause or bilateral oophorectomy at enrolment were considered premenopausal regardless of age, hysterectomy or use of hormonal replacement therapy until they reached the age of 50, at which time they were considered postmenopausal
Ho et al.	2010	No change in menstruation pattern
Hsu et al.	2006	Unclear
Hu et al.	2016	Unclear
Hunter et al.	1996	Unclear
Iida et al.	2011	Unclear
Ilich-Ernst et al.	2002	Unclear
Ito et al.	1994	Normal and regular menstrual cycle
Jaff et al.	2015	STRAW: Regular menstrual cycle and/or with subtle changes to flow or length
Janssen et al.	2008	Bleeding in the last month
Jasienska et al.	2005	Unclear
Jeenduang et al.	2014	Unclear
Jeon et al.	2011	The women said that they were premenopausal
Jurimae et al.	2007	Regular menstrual periods
Kadam et al.	2010	Women above 40 years of age with regular menstruation
Kang et al.	2016	Unclear
Kaufer-Horwitz et al.	2005	Menstrual cycles are regular (25-28 x3) and without any recent changes
Kim et al.	2007	Unclear
Kim et al.	2012	Unclear

(continued)

Study	Year	Premenopause definition
Kim et al.	2013	Unclear
Kim et al.	2016	Unclear
Kirchengast et al.	1996	Regular menstrual cycles
Kirchengast et al.	1998	Regular and probably ovulatory menstrual cycles and E2 >25pg/ml and FSH levels <40 mIV/ml typical of the fertile phase of life
Knapp et al.	2001	Unclear
Koh et al.	2008	Not experiencing menopause on the basis of regularity of menstrual cycles
Konrad et al.	2011	Unclear
Kontogianni et al.	2004	Unclear
Konukoglu et al.	2000	Unclear
Koskova et al.	2007	All of the reproductive years before the onset of menopause i.e. before the cessation of reproductive functions, but with the first endocrine signs of climacterium which start around the age of 40
Kotani et al.	2011	Unclear
Kraemer et al.	2001	Unclear
Kuk et al.	2005	Unclear
Laitinen et al.	1991	Unclear
Lee et al.	2009	Have at least one menstrual period within the 3 months before enrollment
Lejskova et al.	2012	Less than 33 postmenstrual days
Leon-Guerrero et al.	2017	Women who were still menstruating at the reference date
Ley et al.	1992	Regular menstrual cycles and no menopausal symptos
Lin et al.	2006	Unclear
Lindquist et al.	1980	Those who had menstruations during the last month
Lindsay et al.	1992	Unclear
Liu-Ambrose et al.	2006	Menstruation occurred in the last 12 months
Lovejoy et al.	2008	Unclear
Lyu et al.	2001	Unclear
Macdonald et al.	2005	Regular menses
Maharlouei et al.	2013	The entire period of a women's life between menarche and perimenopause (menopausal transition) and terminates with the commencement of menopause
Malacara et al.	2002	Women with regular cycles
Manabe et al.	1999	Unclear
Manjer et al.	2001	No cessation of menses and no hormonal medication use
Mannisto et al.	1996	Unclear
Martini et al.	1997	Unclear
Marwaha et al.	2013	Women <= 50
Matsushita et al.	2003	Regular menstrual cycling
Matsuzaki et al.	2017	Unclear

(continued)

Study	Year	Premenopause definition
Matthews et al.	1989	Menstrual bleeding within the three previous months
Mesch et al.	2006	Women with regular cycles
Meza-Munoz et al.	2006	Women 25-45 years of age, with normal regular cycles and without hormone contraception
Minatoya et al.	2014	Unclear
Mo et al.	2017	Unclear
Muchanga et al.	2014	Women who reported unchanged or irregular menstrual pattern
Muti et al.	2000	Unclear
Nitta et al.	2016	Unclear
Noh et al.	2013	Not postmenopausal
Nordin et al.	1992	Unclear
Ohta et al.	2010	Unclear
Oldroyd et al.	1998	Unclear
Pacholczak et al.	2016	Unclear
Park et al.	2012	Unclear
Park et al.	2017	Still cycling, hysterectomy, ablation, or embolization, and <55 years old.<br>Ovarian suppressing drugs or contraception that eliminated menstrual flow and <55 years of age
Pavicic et al.	2010	Women with regular periods
Pavlica et al.	2013	Unclear
Phillips et al.	2008	Unclear
Polesel et al.	2015	Ongoing menstrual cycle
Pollan et al.	2012	Regular menstruation
Portaluppi et al.	1997	Regular menstrual periods and serum FSH <50
Priya et al.	2013	Unclear
Rantalainen et al.	2010	Women below 35 years of age and assumed to be premenopausal
Razmjou et al.	2018	Two menstruations in the last 3 months, no increase in cycle irregularity in the 12 months before testing and FSH <30IU/L
Reina et al.	2015	Unclear
Revilla et al.	1997	Menstrual histories indicated current and prior menstrual regularity 11-13 cycles per year
Revilla et al.	1997	Menstrual histories indicated current and prior menstrual regularity 11-13 cycles per year
Rice et al.	2015	Women who stated that they had not undergone menopause
Rico et al.	2001	Menstrual histories indicated current and prior menstrual regularity 11-13 cycles per year
Rico et al.	2002	Menstrual histories indicated current and prior menstrual regularity 11-13 cycles per year
Roelfsema et al.	2016	Regular periods
Rosenbaum et al.	1996	Menstruating regularly
Salomaa et al.	1995	Regular menstrual cycles
Sarrafzadegan et al.	2013	Unclear
Schaberg-Lorei et al.	1990	Unclear
Schwarz et al.	2007	WHO definition i.w. whole reproductive period up until menopause

(continued)

Study	Year	Premenopause definition
Shakir et al.	2004	Women who still had regular menstruation
Sherk et al.	2011	Unclear
Shibata et al.	1979	Unclear
Sieminska et al.	2006	Regular menstrual cycles
Skrzypczak et al.	2005	Women who were menstruating
Skrzypczak et al.	2007	Women who were menstruating
Soderberg et al.	2002	Regular menstruation
Son et al.	2015	Regular menstrual periods
Soreca et al.	2009	Had menstruated in the previous 3 months
Soriguer et al.	2009	Unclear
Staessen et al.	1989	Unclear
Suarez-Ortegon et al.	2012	Unclear
Suliga et al.	2016	Unclear
Sumner et al.	1998	Unclear
Tanaka et al.	2015	Regular menstruation
Thomas et al.	2000	Unclear
Torng et al.	2000	Unclear
Toth et al.	2000	The occurrence of two menses in the 3 months preceding testing, no increase in cycle irregularity in the 12 months preceeding testing and FSH < 30
Tremollieres et al.	1996	Unclear
Trikudanathan et al.	2013	Unclear
Van-Pelt et al.	1998	Normal or regular menstruation
Veldhuis et al.	2016	Unclear
Wang et al.	2012	Unclear
Wang et al.	2006	Unclear
Wang et al.	2012	45 <= age <= 55
Wee et al.	2013	Women with regular menses during the 2 years preceeding recruitment into the study
Williams et al.	1997	12-50 year olds having periods
Wing et al.	1991	Menstruated in the past 3 months
Xu et al.	2010	Regular menstruation without significant variation between the menses or in the number of days of menstrual bleeding in each period over the course of the preceeding year
Yamatani et al.	2013	Unclear
Yannakoulia et al.	2007	Regular menses
Yoldemir et al.	2012	Any women with more than 8 menses in the last year were considered to be premenopausal
Yoo et al.	2012	Unclear
Yoo et al.	1998	Regular menstrual cycle
Yoshimoto et al.	2011	Unclear

(continued)

Study	Year	Premenopause definition
Zhong et al.	2005	Unclear
Zhou et al.	2010	Regular menstruation
Zhou et al.	2015	Unclear
Zivkovic et al.	2011	Unclear
