# Supplementary material for: A review of menopause nomenclature
Source: Reprod Health. 2022 Jan 31;19:29. doi: 10.1186/s12978-022-01336-7 (PMC8805414; doi:10.1186/s12978-022-01336-7)
Supplement: Supplementary file 2 — Additional file 2: Table 2. Postmenopause definition. [file 12978_2022_1336_MOESM2_ESM.pdf]

Additional Table 2: Postmenopause definition.

**Study**	**Year**	**Postmenopause definition**
Abate et al.	2014	Women whose menstrual cycles had stopped for at least 2 years, but no more than 7 years
Abdulnour et al.	2012	Based on the final menstrual period and confirmed by 12 months of amenorrhea
Abildgaard et al.	2013	Based on the final menstrual period and confirmed by 12 months of amenorrhea and FSH >20IU/L
Adams-Campbell et al.	1996	Unclear
Agrinier et al.	2010	Based on the final menstrual period and confirmed by 12 months of amenorrhea
Aguado et al.	1996	Unclear
Akahoshi et al.	2001	Two years after the final menstrual period
Albanese et al.	2009	One to five years since menopause (greater than 12 months from their last menstrual bleeding)
Allali et al.	2009	Unclear
Aloia et al.	1995	Unclear
Amankwah et al.	2013	Stopped menstruation for 1 year and age was 50 or more
Amarante et al.	2011	One year or more of amenorrhea after the age of 40 years
Amiri et al.	2014	Time of cessation of menstrual periods for 12 consecutive months, not due to surgery or any other biological or physiological causes
Angsuwanthana et al.	2007	Women with bilateral oophorectomy, for hysterectomised women without bilateral oophorectomy (FSH >40) and for natural menopause,if younger than 45 the women had amenorrhea for greater than 1 year and FSH > 40 and if older than 45 years, the women had amenorrhea for >1 year
Armellini et al.	1996	Unclear
Arthur et al.	2013	Ceased menstruation for at least one year
Aydin et al.	2010	12 months past final menses
Ayub et al.	2006	Unclear
Bancroft et al.	1996	Women whose last menopausal period occurred more than 12 months previously
Bednarek-Tupikowska et al.	2006	Unclear
Bell et al.	2007	A decision tree including - older than 55, a stated age at menopause, no menstrual bleeding, potential use of hormone contraception and FSH levels
Ben-Ali et al.	2016	Women with natural menopause whose current age was >= 1 year from the final menstrual period and who did not receive hormone replacement therapy
Ben-Ali et al.	2014	Women with natural menopause whose current age was >= 1 year from the final menstrual period and who did not receive hormone replacement therapy
Ben-Ali et al.	2011	At least 12 consecutive months of amenorrhea with no other medical cause
Berg et al.	2004	At least 1 year of spontaneous amenorrhea
Berge et al.	1994	Natural menopause occurred at least one year ago
Berger et al.	1995	Spontaneously postmenopausal with secondary amenorrhea for a minimum of 6 months, elevated gonadotropin levels and reduced oestrogen levels
Berstad et al.	2010	FMP occurred >12 months before the reference date and had not used hormone therapy before or during the 12month interval after the FMP, or if she had undergone bilateral oophorectomy
Bhagat et al.	2010	Reporting last menses to be at least 12 months previously (i.e. no menstruation for at least 1 year)
Bhurosy et al.	2013	Those who had their last menstrual bleeding at least one year before

(continued)

Study	Year	Postmenopause definition
Blumenthal et al.	1991	No menses in the 12 months prior to participating in the study and had FSH levels greater than 40
Bonithon-Kopp et al.	1990	Passed the menopause and their periods has stopped spontaneously more than three months before examination
Caire-Juvera et al.	2008	No menstrual cycle within the past 12 months or FSH level between 22 and 138 mIU/mL
Campesi et al.	2016	At least 1 year without menstrual cycle
Carr et al.	2000	12 months with no bleeding or spotting after age 35, not due to use of exogenous hormones
Castracane et al.	1998	Unclear
Catsburg et al.	2014	Unclear
Cecchini et al.	2012	Women who reported that both of her ovaries were removed or if she indicated that her menstrual periods had stopped for at least 12 months
Cervellati et al.	2009	Amenhorrea for longer than 11 months
Chain et al.	2017	Cessation of natural menses for >= 12 months
Chang et al.	2000	Amenorrhea greater than 12 months duration
Cho et al.	2008	Absence of menses for 12 consecutive months
Cifkova et al.	2008	FMP had occurred more than 365 days before the interview with FSH levels > 40IU/L
Copeland et al.	2006	One year of menses cessation and FSH levels >30 mIU/ml
Cremonini et al.	2013	Periods of amenorrhea longer than 12 months
Cui et al.	2007	At least 12 months of amenorrhea resulting from the permanent cessation of ovarian function
D'haeseleer et al.	2011	12 consecutive months of amenorrhea
Da Camara et al.	2015	Absence of menses for over one year
Dallongeville et al.	1995	No menstruation during the 12 months before examination
Dancey et al.	2001	More than 55 years of age
Davis et al.	1994	No menstrual cycles or periods
De Kat et al.	2017	Date of last menstruation was more than 1 year before the visit
Den Tonkelaar et al.	1990	Menstruation had stopped spontaneously more than 12 months before
Dmitruk et al.	2018	Menostasis was longer than 12 months
Donato et al.	2006	Women presenting with 12 months or more of amenorrhea, or as a result of medical interventions, such as bilateral oophorectomy
Douchi et al.	1997	Unclear
Douchi et al.	2002	No menstruation for 12 months before the investigation
Douchi et al.	2007	No menstruation for 12 months before the investigation
Dubois et al.	2001	Women who were amenorrhoeic for at least 12 months
Engmann et al.	2017	Self-reported as postmenopausal (natural or both ovaries removed), or age 55+ or current HRT use
Ertungealp et al.	1999	Unclear
Feng et al.	2008	Menstruation stopped for at least 12 months
Ford et al.	2005	Absence of a menstrual bleed for a 12-month period or a history of bilateral oophorectomy
Formica et al.	1995	Unclear
Franklin et al.	2009	No menses for 1 year

(continued)

Study	Year	Postmenopause definition
Friedenreich et al.	2007	Not having had any menses over the past 12 months or if they had a bilateral oophorectomy or if they were using HRT and over the age of 55
Friedenreich et al.	2002	Stopped menstruation for 1 year and age was 50 or more
Fu et al.	2011	Complete natural cessation of menses for more than 12 months
Fuh et al.	2003	No menstruation within the previous 12 months
Gambacciani et al.	1999	No menstruation for 6 or more months prior to the study
Genazzani et al.	2006	No menstruation for 6 or more months prior to the study
Ghosh et al.	2008	Reporting last menses to be at least 12 months previously (i.e. no menstruation for at least 1 year)
Ghosh et al.	2010	Reporting last menses to be at least 12 months previously (i.e. no menstruation for at least 1 year)
Gram et al.	1997	Stopped menstruation for at least 1 year
Guo et al.	2015	Women older than 53 years at recruitments and/or had had both ovaries removed were categorized as postmenopausal
Gurka et al.	2016	Women who had not had a period in the past 2 years and did not have durgical removal of ovaries or uterus
Hadji et al.	2000	Women with a hysterectomy and bilateral oophorectomy and/or no menstrual periods in the year preceding their examination and/or a serum FSH level of <10pg/ml
Hagner et al.	2009	FMP more than 365 days before examination
Han et al.	2006	Menses had ceased permanently and naturally or bilateral oophorectomy, hysterectomy without the removal of ovaries and older than 50
Harting et al.	1984	Unclear
He et al.	2012	Menstruation had naturally stopped for at least one year without bilateral oophorectomy, simple hysterectomy, hormone therapy or currently pregnant
Hirose et al.	2003	Unclear
Hjartaker et al.	2005	Only women who reported natural menopause or bilateral oophorectomy or over the age of 50
Ho et al.	2010	At least 12 months since the last menses
Hsu et al.	2006	Unclear
Hu et al.	2016	Unclear
Hunter et al.	1996	Absence of menses for one year
Iida et al.	2011	Unclear
Ilich-Ernst et al.	2002	Unclear
Ito et al.	1994	Absence of menstrual period for at least 6 months
Jaff et al.	2015	STRAW: 0-6 years after the final menstrual period
Janssen et al.	2008	Bleeding was more than 12 months ago
Jasienska et al.	2005	Unclear
Jeenduang et al.	2014	Absence of menstruation for a preceding 12 months minimum
Jeon et al.	2011	Cessation of menstruation for at least 1 year
Jurimae et al.	2007	Postmenopausal for >1 year but <7 years
Kadam et al.	2010	Permanent cessation of menstrual periods that occurs naturally or is induced by surgery in accordance with the definition by WHO

(continued)

Study	Year	Postmenopause definition
Kang et al.	2016	Unclear
Kaufer-Horwitz et al.	2005	>12 months of amenorrhea
Kim et al.	2007	A woman with natural menopause whose current age was >= 1 year than her age of menopause who did not receive HRT
Kim et al.	2012	Unclear
Kim et al.	2013	Cessation of menstruation for at least 1 year, with a FSH level greater than 30
Kim et al.	2016	Unclear
Kirchengast et al.	1996	Menopause had occurred spontaneously
Kirchengast et al.	1998	Spontaneous menstrual bleeding had occurred at least 1 year before the investigation with estradiol <25pg/ml and FSH >40miV/ml
Knapp et al.	2001	Unclear
Koh et al.	2008	Had menses >1 year before the study
Konrad et al.	2011	Cessation of menses for at least 12 consecutive months
Kontogianni et al.	2004	Absence of menses for more than 6 months and by elevated serum FSH levels (FSH>40 U/L)
Konukoglu et al.	2000	Absence of menstruation for at least 6 months and a serum concentration of FSH of >40 IU/ml
Koskova et al.	2007	The last physiological endometrial bleeding and can be assessed retrospectively after 1 year of absence of bleeding
Kotani et al.	2011	Cessation of menses for a period of 12 months or longer
Kraemer et al.	2001	Unclear
Kuk et al.	2005	Unclear
Laitinen et al.	1991	Unclear
Lee et al.	2009	The lack of menstrual periods for at least 12 consecutive months
Lejskova et al.	2012	More than 365 postmenstrual days
Leon-Guerrero et al.	2017	Women whose most recent preiod was more than 12 months before the reference date
Ley et al.	1992	Amenorrhea and elevated gonadotrophin concentrations
Lin et al.	2006	Menopause is defined as the absence of menstruation for 12 consecutive months, which is not due to surgical resection of the uterus or ovaries
Lindquist et al.	1980	Those who had no menstruation during a period fo >= 6 months before the study
Lindsay et al.	1992	Unclear
Liu-Ambrose et al.	2006	No menstruation had occurred in the last 12 months
Lovejoy et al.	2008	No menstrual cycles in the past year and FSH>30mIU/ml
Lyu et al.	2001	Having no menstrual bleeding for >= 1year
Macdonald et al.	2005	Women who had ceased menstruating for at least 1 year and had never taken HRT
Maharlouei et al.	2013	The cessation of menses for a minimum of 12 months and encompasses the entire period in a women's life that takes place after her last period (menopause)
Malacara et al.	2002	Women with previous regular cycles, without menses in previous 12 months
Manabe et al.	1999	Unclear
Manjer et al.	2001	Women whose menses have ceased or are taking HRT
Mannisto et al.	1996	Unclear

(continued)

Study	Year	Postmenopause definition
Martini et al.	1997	No menstrual bleeding for at least 6 months preceeding
Marwaha et al.	2013	Woman >= 50
Matsushita et al.	2003	Unclear
Matsuzaki et al.	2017	Unclear
Matthews et al.	1989	Stopped menstruating for at least 12 months
Mesch et al.	2006	Women with 1 year of spontaneous amenorrhea
Meza-Munoz et al.	2006	Women older than 48 with at least 1 year since their last menses, with previously regular cycles
Minatoya et al.	2014	Unclear
Mo et al.	2017	Unclear
Muchanga et al.	2014	Women who reported their last menses to be at least 12 months prior to this study
Muti et al.	2000	The absence of menstrual bleeding for at least 12 months
Nitta et al.	2016	Unclear
Noh et al.	2013	No menstruation for the last 12 months and met one of the following conditions: (1) reported natural menopause, (2) received bilateral oophorectomy, (3) had ever taken HRT, (4) had an FSH level >30 or (5) was older than 55
Nordin et al.	1992	Unclear
Ohta et al.	2010	Unclear
Oldroyd et al.	1998	Unclear
Pacholczak et al.	2016	No menses for 12 months or bilateral oophorectomy or hysterectomy
Park et al.	2012	Unclear
Park et al.	2017	No menstrual periods in the last 12 months or had both ovaries removed, chemotherapy/radiation that stopped periods, hysterectomy, ablation, or embolization and >55 years of age, ovarian suppressing drugs or contraception that eliminated menstrual flow and >=55 years of age
Pavicic et al.	2010	One year of amenorrhoea
Pavlica et al.	2013	Unclear
Phillips et al.	2008	Absence of menstruation for at least one year
Polesel et al.	2015	Amenorrhea for more than 1 year and FSH and LH concentrations higher than 30l
Pollan et al.	2012	Absence of menstruation in the last 12 months
Portaluppi et al.	1997	Women with last menstrual period at least 12 months before they entered and FSH >50
Priya et al.	2013	At least 1 year of cessation of menses
Rantalainen et al.	2010	Self-report
Razmjou et al.	2018	Based on the final menstrual period and confirmed by 12 months of amenorrhea.
Reina et al.	2015	Unclear
Revilla et al.	1997	No menstrual periods for at least 12 months
Revilla et al.	1997	No menstrual periods for at least 12 months
Rice et al.	2015	Women who had undergone menopause defined as the permanent cessation of periods for more than 12 months
Rico et al.	2001	No menstrual period for at least 12 months and serum FSH levels >30
Rico et al.	2002	No menstrual period for at least 12 months and serum FSH levels >30

(continued)

Study	Year	Postmenopause definition
Roelfsema et al.	2016	Based on medical history and FSH >30
Rosenbaum et al.	1996	No menstruation for at least 7 years
Salomaa et al.	1995	No menstrual cycles
Sarrafzadegan et al.	2013	Unclear
Schaberg-Lorei et al.	1990	Unclear
Schwarz et al.	2007	12 consecutive months of amenorrhea and is not due to causes and procedures such as hysterectomy that would be associated with cessation of menses
Shakir et al.	2004	Women whose menstruation had ceased more than 12 months ago
Sherk et al.	2011	Unclear
Shibata et al.	1979	Unclear
Sieminska et al.	2006	Amenorrhoea for at least 1 year
Skrzypczak et al.	2005	Women whose last menstruation occurred earlier than 12 months before the participation of the study
Skrzypczak et al.	2007	Women whose last menstruation occurred earlier than 12 months before the participation of the study
Soderberg et al.	2002	More than 6 months since last menstruation
Son et al.	2015	The period after 12 consecutive months of amenorrhea
Soreca et al.	2009	Unclear
Soriguer et al.	2009	6 months of amenorrhea
Staessen et al.	1989	Definitive cessation of periods or cessation of periods following a gynecological operation
Suarez-Ortegon et al.	2012	Unclear
Suliga et al.	2016	Women with amenorrhea for at least 12 months
Sumner et al.	1998	Women who had not menstruated for 1 year OR women with hysterectomy IF she was older than 55
Tanaka et al.	2015	Absence of menstruation for the last 2 years
Thomas et al.	2000	Documented bilateral oophorectomy or a duration longer than 6 months without a menstrual period
Torng et al.	2000	Women with secondary amenorrhea of at least 1 year
Toth et al.	2000	Absence of menses for at least 6 months and a FSH level >30
Tremollieres et al.	1996	Amenorrhea of >=6 months and estradiol <20 and FSH > 30
Trikudanathan et al.	2013	Periods stopped for 1 year or more
Van-Pelt et al.	1998	FSH >30 and absence of menses
Veldhuis et al.	2016	FSH >30 and E2 <50pg/ml
Wang et al.	2012	Unclear
Wang et al.	2006	No menses for at least 12 months
Wang et al.	2012	Age >= 55
Wee et al.	2013	Cessation of menses for at least 12 months prior to the study
Williams et al.	1997	>= 40 years old and not having periods
Wing et al.	1991	Stopped menstruating for at least 12 months, did not have surgical menopause or HRT in the last year
Xu et al.	2010	Have not experienced any menstrual flow for a minimum of 12 months and FSH >30
Yamatani et al.	2013	Amenorrhea for at least 12 months, and FSH > 30 and E2 lower than 20

(continued)

Study	Year	Postmenopause definition
Yannakoulia et al.	2007	Women who had ceased menstruating for at least 12 months
Yoldemir et al.	2012	Absence of menstruation for the preceeding 12 months or more
Yoo et al.	2012	At least 12 months of amenorrhea resulting in permanent cessation of ovarian function
Yoo et al.	1998	Women whose last menstrual cycle occurred at least six months prior to the survey and aged over 35
Yoshimoto et al.	2011	Unclear
Zhong et al.	2005	Unclear
Zhou et al.	2010	Having 12 consecutive months of amenorrhea with no other causes
Zhou et al.	2015	Women who reported menses had ceased for 1 year or more
Zivkovic et al.	2011	Women less than 2 years from menopause, where menopause is 12 months of amenorrhea
